# Super super obesity with a BMI of 98 kg/m^2^: a case report

**DOI:** 10.1093/jscr/rjad690

**Published:** 2024-01-10

**Authors:** Ragad I Al Jazzar, Nayef Alzahrani, Mustafa M Eltayeb, Ahmed K Satti, Hussain Omar, Arief Arrowaili

**Affiliations:** College of Medicine, King Saud bin Abdulaziz University for Health Sciences, Riyadh, Saudi Arabia; Department of Surgery, King Abdulaziz Medical City, Ministry of National Guard Health Affairs, Riyadh, Saudi Arabia; Department of Surgery, Riyadh Care Hospital, Riyadh, Saudi Arabia; Department of Surgery, Riyadh Care Hospital, Riyadh, Saudi Arabia; Department of Surgery, Riyadh Care Hospital, Riyadh, Saudi Arabia; College of Medicine, Al Imam Mohammad Ibn Saud Islamic University, Riyadh, Saudi Arabia

**Keywords:** laparoscopy, sleeve gastrectomy, super super obesity

## Abstract

Standards of care in regards to super-super obese patients are yet to be evolving due to the sparsity of this weight category along with the high morbidity and mortality rates attributed to it. We report a successful case of laparoscopic sleeve gastrectomy for a 35 years old lady with a body mass index (BMI) of 98 kg/m^2^.

## Introduction

Obesity is an ever growing problem of the 21st century. Carrying multiple health and economic burdens upon its affected individuals. The World Health Organization defined overweight and obesity as an excess fat build up that would carry risks on the overall health. Children and adolescence between the ages of 5 and 19 years old showed a 4-fold increase in developing obesity since 1975 [1]. The Saudi Ministry of Health reported 20% of its citizens were obese, with a higher prevalence in females 21%, in 2019. The same report revealed that inadequate physical activity was estimated to be ~80% in the Saudi population [2]. The high economic impact of obesity is attributed to its chronic span of health-related complications with significant morbidity and mortality rates [3]. Bariatric surgeries are well-established methods of weight reduction and improvement and/or elimination of obesity-related comorbidities. Body mass index is the parameter of choice for the assessment of weight status. Morbid obesity was addressed as a body mass index (BMI) ≥ 40 kg/m^2^, super obesity as a BMI ≥ 50 kg/m^2^, and super super obesity as a BMI ≥ 60 kg/m^2^.

We report a case of a super-super obese patient that underwent a laparoscopic sleeve gastrectomy (LSG) with no peri nor postoperative complications.

## Presentation

A 35 years old female, known case of hypothyroidism, presented to the bariatric clinic with the intention of seeking a solution to her weight gain. The patient has tried reducing her weight by altering daily habits in terms of diet and implementing physical activity in her schedule. Nevertheless, was unmotivated to continue due to the minimal weight loss that was observed over a 6 months period. After assessing her body profile, which was as follows: weight: 221 kg, height:150 cm, BMI: 98 kg/m^2^, waist circumference was 200 cm. A multidisciplinary team was held to discuss the case including: internal medicine, psychiatry, and a dietician. We suggested two bariatric surgery options: one anastomosis mini gastric bypass (OAGB) or LSG. The decision was opted for the latter one, as a bridging surgery then proceed to the OAGB for reaching an optimal weight loss.

The patient was admitted and underwent LSG in 2021.

### Positioning

The patient was positioned supine then moved to reverse Trendelenburg position and a troop intubation pillow was used, intubated using an orogastric tube with a direct laryngoscope (DL) with no special measurements offered. A veress needle was inserted through a small incision in the left subcostal area (Palmer’s point) and pneumoperitoneum was achieved. A 12 mm port was inserted using an optical trocar, no injuries were made, under direct visualization, 2 more 10 mm assisting ports were inserted superio-laterally to the main port. Liver retractor was not used according to the surgeons preference, and a total of three ports were used.

### Gastric mobilization

The greater omentum was cautiously dissected from the greater curvature of the stomach using ultrasonic scissors (harmonic). Dissecting the short gastric arteries close to the stomach serosal layer moving superiorly toward the fundus, reaching the cardia, left crus, and the fat pad. Once it was released, the dissection was directed toward the pylorus sparing ~4 cm before reaching it.

### Gastrectomy

A 36-French bougie calibration tube was placed reaching the pylorus. A wide incisura angle was insured during the stapling to preserve the anatomical shape of the remaining stomach pouch. Five linear staplers using Endo GIA. Routine leak test was performed and the specimen was then removed via the left port. Sleeve gastrectomy was done uneventfully with a total operation time of 25 minutes. Later, the patient was shifted to the recovery room for an hour for monitoring. After spending a day in the ward, the patient was discharged with no major postoperative complications nor ICU admission. Currently, the patient is followed up for 2 years and revealed a weight loss of 120 kg, BMI 53.33 of kg/m^2^, a percentage of excess weight loss (%EWL) was 45.4%.

## Discussion

Obesity is associated with numerous chronic comorbidities, including diabetes, hypertension, dyslipidemia, and obstructive sleep apnea. The approximate costs of health complications related to obesity in the private sector are as follows: heart failure, dyslipidemia, and diabetes mellitus type 2, 32 020 SAR, 26 369 SAR, 23 271 SAR per person per year, respectively. In the same study, it was estimated that ~6 million Saudis in their early 20s up to late 60s are within a BMI range of 30–50 kg/m^2^. Furthermore, the total 10 years economic savings if they lost 15% of their weight would nearly be 63.7 billion SAR [3]. Jeong et al. has reported a similar case of a patient with a BMI of 85.8 kg/m^2^ that underwent LSG with favorable outcomes, 2 years follow up revealed a %EWL was 43.2% [4]. Another study compared the clinical outcomes of one anastomosis gastric bypass versus sleeve gastrectomy in super-super-obese patients with no difference in conversion to open nor mortality rates which was 0 in both. Regarding comorbidities both groups showed similar remission or improvement in diabetes mellitus type 2, sleep apnea, and orthopedic comorbidities with an exception in hypertension where remission rates were higher in OAGB compared with LSG. A 12 months follow up revealed a weight loss of 65 and 59%, respectively, in OAGB and LSG. Unfortunately, more patients complained of inadequate weight loss or weight regain in the LSG group compared with OAGB [5]. In our case, the patient did not have comorbid diseases apart from increased fatigability, muscle pain and decreased exercise tolerance due to increased weight. Therefore, our only measurement was the %EWL, which was 45.4% in a 2 year period postoperatively with no weight regain. OAGB was planned to be the next step in management, but the patient was hesitant to further proceed with the second surgery. Our patient was intubated using the conventional DL and a troop intubation pillow by a well-trained Anesthesiologist which was done smoothly without the need of video assisted intubation. Video laryngoscope (VD) was used in a case of an LSG in a patient with a BMI of 103 kg/m^2^ which was reported to be an effective and efficient way of intubation for obese patients [6]. No further peri-anesthesia measurements were specifically undertaken in comparison to patients with lower BMI. Up to our knowledge, this is the first case reporting such massive BMI treated successfully with sleeve gastrectomy.

## Conclusion

We reported a first successful case of a patient with a BMI of 98 kg/m^2^ in the Saudi population that underwent an LSG in a standardized manner without the need of peri or postoperative special management. Nonetheless, super super obese individuals can undergo LSG with standardized measures with a good outcome under well-trained surgeons in a highly equipped secondary (private) hospital.
